# Complete genome sequence of *Bacillus subtilis* NA05 (=NBRC 116153), a phosphate-solubilizing bacterium isolated from crop field soil

**DOI:** 10.1128/mra.00154-24

**Published:** 2024-05-20

**Authors:** Kerui Guo, Takashi Kunito, Shigeto Otsuka

**Affiliations:** 1 Graduate School of Agricultural and Life Sciences, The University of Tokyo, Tokyo, Japan; 2 Faculty of Science, Shinshu University, Matsumoto, Nagano, Japan; 3 Collaborative Research Institute for Innovative Microbiology, The University of Tokyo, Tokyo, Japan; Indiana University, Bloomington, Bloomington, Indiana, USA

**Keywords:** soil microbiology, phosphate, genomes

## Abstract

We report the complete genome sequence of the phosphate-solubilizing bacterium *Bacillus subtilis* NA05 (=NBRC 116153), consisting of a circular chromosome of ~3.8 M bp and two circular plasmids. The data presented here provide further insight into the genetic and functional potential of *B. subtilis* and the mechanism of phosphate solubilization.

## ANNOUNCEMENT

*Bacillus* species contain plant pathogen-antagonistic (1), plant growth-promoting (2), phosphate-solubilizing populations, etc. Here, we report the complete genome information of phosphate-solubilizing *Bacillus subtilis* strain NA05 (NBRC 116153).

*B. subtilis* NA05 was isolated from crop field soil (Andosol) collected from the Institute for Sustainable Agro-ecosystem Services, The University of Tokyo, Japan, according to our previous study (3) using a medium with glucose and iron(III) phosphate as each of sole carbon and phosphorus sources. The strain has been continuously sub-cultivated on the same medium. DNA was extracted from the cultivated bacteria using a Genomic-Tip 20/G (Qiagen, Hilden, Germany) according to the manufacturer’s protocol. Complete genome analysis was commercially conducted by Seibutsu Giken (Kanagawa, Japan) as follows. Library preparations were performed using SMRTbell Express Template Prep Kit 2.0 and SMRTbell gDNA Sample Amplification Kit (PacBio, Menlo Park, CA, USA) according to the manufacturer’s protocol. DNA fragmentation was not performed. As low-molecular-weight DNA was recognized, they were removed using Short Read Eliminator XS (PacBio). Polymerase complexes of the libraries were formed as per the manual using Binding Kit 2.2 (PacBio) and sequenced on Sequel IIe (PacBio) using Sequel II 2.0 chemistry. CCS reads with an average quality value of less than 20 per read were removed and designated as HiFi reads. No error was corrected. UltraLowPCR adaptor was removed from HiFi reads using adaptor trimming lima (version 2.6.0). HiFi reads exceeding 1,000 bp were *de novo* assembled using Flye (v.2.9.1-b1780) with the default parameters. Ultra-LowPCR adapters and PCR duplicate reads were removed using lima (version 2.6.0) and pbmarkdup (version 1.0.2). The genome coverage was 39×, read *N*_50_ was 3,886,763 bp, the number of reads was 28,178, and the number of contigs was 1 for each of a chromosome and two plasmids. The integrity of the assembled genome data was assessed using CheckM (version 1.2.2).

The chromosome of 3,886,763 bp and the two plasmids of 128,740 and 20,109 bp are all circular, with G + C content of 43.8%, 43.6%, and 44.4%, respectively. The public genome annotation with the DFAST v. 1.6.0 (4) revealed 3,977 coding sequences, 102 pseudogenes, 82 tRNA genes, and 25 rRNA genes for this strain, which are comparable with the type strain of *B. subtilis* ATCC 6051^T^ that has 4,250 coding sequences, 79 pseudogenes, 87 tRNA genes, and 30 rRNA genes annotated by DFAST.

According to the annotations, *B. subtilis* NA05 has nitric oxide reductase activation proteins, NorD and NorQ. *Bacillus* species/strains are generally capable of denitrification (5), but this strain is only capable of nitric oxide reduction included in denitrification steps. The strain also possesses 100% of the genes required for dissimilatory nitrate reduction. The strain does not possess acid phosphatase but possesses alkaline phosphatase and phytase, which is the same as the type strain ATCC 6051^T^.

## Data Availability

The complete genome sequences of *B. subtilis* NA05 are available from DDBJ/EMBL-Bank/GenBank under the accession numbers AP028964, AP028965, and AP028966, with the raw sequence reads, the BioProject, and the BioSample under accession numbers DRA017954, PRJDB16794, and SAMD00650365, respectively.
